# The Antimicrobial Stewardship Approach to Combating *Clostridium Difficile*

**DOI:** 10.3390/antibiotics4020198

**Published:** 2015-06-17

**Authors:** Eric Wenzler, Surafel G. Mulugeta, Larry H. Danziger

**Affiliations:** 1 College of Pharmacy, University of Illinois at Chicago, Chicago, IL 60612, USA; E-Mails: wenzler@uic.edu (E.W.); smulug2@uic.edu (S.G.M.); 2 College of Medicine, University of Illinois at Chicago, Chicago, IL 60612, USA

**Keywords:** antimicrobial stewardship, *Clostridium difficile*, restriction, diarrhea, rapid diagnostics, treatment, infection control

## Abstract

*Clostridium difficile* remains a major public health threat and continues to contribute to excess morbidity, mortality and healthcare costs. Antimicrobial stewardship programs have demonstrated success in combating *C. difficile*, primarily through antibiotic restrictive strategies. As the incidence and prevalence of *C. difficile* associate disease continues to increase both in the hospital and community setting, additional stewardship approaches are needed. This manuscript reviews stewardship interventions that have been successful against *C. difficile* associated disease and proposes future tactics that antimicrobial stewardship programs may employ to develop a more global approach to combat this difficult pathogen.

## 1. Introduction 

“A pessimist sees the difficulty in every opportunity; an optimist sees the opportunity in every difficulty.”—Winston Churchill

In 2013, the Centers for Disease Control and Prevention (CDC) classified *Clostridium difficile* (CD) as a pathogen that represented an immediate threat to public health and one that requires urgent and aggressive action [1]. CD associated disease (CDAD) is responsible for approximately 250,000 infections and 14,000 deaths per year in the United States, accounting for $1 billion in excess medical costs annually. Despite increased awareness and action to combat CDAD, CD is now the most frequently reported nosocomial pathogen. Of particular concern is the recent spread of CD to the community setting, often without traditional risk factors for CDAD [2]. Along with the continued increase in the overall tonnage of antimicrobial use in both animals and humans, hypervirulent antibiotic-resistant strains such as the North American pulsed-field gel electrophoresis type (NAP1) strain also continue to become more prevalent and contribute to the increasing severity of CDAD. 

It is known that antibiotic exposure nearly always precedes CDAD and that commonly prescribed broad-spectrum antibiotics have been particularly implicated [3,4]. Up to 60% of hospitalized patients in the U.S. receive antimicrobials at some point during their admission and are often maintained without de-escalation for up to five days [5]. Antimicrobial stewardship programs (ASPs) are therefore uniquely and adequately positioned to help battle the growing epidemic of CDAD. The very essence of ASPs is to promote the wise and rational use of antimicrobials in order to improve patient outcomes, decrease toxicities, stem the growing tide of antibacterial resistance and prevent untoward consequences of their use, including CDAD. 

The Infectious Diseases Society of America and the Society for Healthcare Epidemiology of America have proposed core strategies for ASPs [6], many of which have proven successful in decreasing the incidence of CDAD [7]. In particular, antimicrobial restriction by ASPs has been one such successful strategy, showing a reduction in the rate of CD infection by up to 77% [8]. Despite these encouraging results, alarmingly only 52% of 398 surveyed hospitals in the U.S. reported regularly using their ASP to combat CDAD [9].

CDAD continues to impose a tremendous burden on the healthcare system, contribute to excess morbidity and mortality and pose a difficult challenge to clinicians. ASPs must meet this challenge by continuing to develop efficient, successful strategies beyond solely labor intensive antimicrobial restrictive interventions. The aim of this review is to evaluate the impact of ASP interventions on CD thus far and to look forward at potential future strategies and opportunities for ASPs to develop a more global approach to combat this difficult pathogen. A systematic literature search was conducted via PubMed and the Cochrane Database of systematic reviews using the search terms “difficile”, “clostridium difficile”, “antimicrobial stewardship”, “stewardship”, “restriction”, “rapid diagnostics”, “bacteriotherapy”, “infection control” and “outpatient” limited to English language and screened for topical relevance. No date limit was placed and we reviewed the reference lists of included articles to identify those not found by the primary search. 

### 1.1. Antibiotic Restrictive Approaches 

Although even appropriate use of antibiotics can lead to development of CDAD, it is estimated that up to 50% of antimicrobials prescribed are done so improperly [1]. Though cephalosporins, clindamycin and fluoroquinolones are most often associated with CDAD, virtually every antibiotic utilized in clinical practice has been implicated [4]. This widespread overuse and misuse of antimicrobials presents a tremendous opportunity for ASPs to optimize patient care. One successful approach for has been the restriction of the availability and/or the prescribing of antibiotics known to pose a high risk of subsequent CDAD acquisition. The following section summarizes examples of ASPs that have implemented this restrictive strategy. 

A recent meta-analysis by Feazel and colleagues describes the impressive effect ASPs can have on the incidence of CDAD [7]. They report the outcomes of several ASPs which have used restricting exposure to certain high risk antibiotics as a method to prevent CDAD among hospitalized adult patients in non-outbreak settings. Overall, implementation of ASPs had a protective benefit with a risk reduction in the rate of CDAD of 52% (pooled risk ratio 0.48; 95% CI: 0.38–0.62). The authors further characterized the ASPs by setting and found that the most significant protective effect was among the geriatric population in acute geriatric wards and hip fracture surgery patients (56% reduction; pooled risk ratio 0.44; 95% CI: 0.35–0.56). This is not surprising as the majority of CD cases occur among the elderly [10], although ASP introduction also had a significant positive impact across the entire hospital setting (37% reduction; pooled risk ratio 0.63; 95% CI: 0.42–0.95). Importantly, when antimicrobial restrictive policies were compared to persuasive policies, *i.e.*, those that aimed at changing prescriber behavior without active restriction, only restrictive policies had a significant protective effect (pooled risk ratio 0.46; 95% CI: 0.38–0.56). When examined by length of intervention and class of antibiotic restricted, longer interventions and those that restricted cephalosporins or fluoroquinolones were shown to be more effective. This analysis highlights antimicrobial restriction as a highly effective method for decreasing CD incidence and provides support for evidenced-based recommendations and guidance for the implementation of ASPs in healthcare institutions interested in reducing their rates of CDAD. 

The results shown by Feazel *et al*. have been previously confirmed in a unique time-series analysis by Aldeyab and colleagues [11]. The authors examined the effect of ASP restriction of second and third generation cephalosporins, fluoroquinolones and clindamycin on the rates of CDAD while accounting for the age-adjusted comorbidity index. The restriction of these high risk antimicrobials led to a statistically significant 0.0047 incidence rate reduction per 100 bed days per month over the six and a half year study period. Multiple other studies not included in the previously mentioned analysis by Feazel *et al*. have also demonstrated the benefit of both clindamycin and cephalosporin restriction, all of which resulted in a reduction in the rates of CDAD [12,13,14,15].

In the majority of these studies, the most common method of restriction is the requirement for antibiotic order approval by an infectious disease (ID) pharmacist or physician. This restriction is commonly achieved by the ID pharmacist or physician carrying an antibiotic approval pager or phone, which can be cumbersome and time and labor intensive. Additionally, it may be difficult to gain buy-in for restrictive strategies from non-ASP physicians, hospital administration and ASPs with limited resources. Despite the effectiveness of antimicrobial restriction, the need for efficiency necessitates the exploration of other non-restrictive strategies aimed at decreasing rates of CDAD. 

### 1.2. Non-Restrictive Approaches 

Non-restrictive ASP approaches to reducing CDAD incidence in the healthcare setting have primarily centered around prospective audit and feedback. Other methods have been evaluated with varying success including education, hospital guideline and policy change, and treatment of asymptomatic CD carriers. ASPs are intimately involved in educating physicians and other colleagues within the healthcare system and implementing and updating institution wide antimicrobial use guidelines. The following section highlights the role that ASPs can play in nonrestrictive strategies with a successful track record at reducing CD rates. 

In the previously discussed meta-analysis by Feazel [7], persuasive interventions, primarily the prospective audit and feedback method, were associated with a 51% reduction in CDAD rates. While review and feedback has been successful in its own right, a change in hospital guidelines and institution wide education has also proven useful. Valiquette and colleagues implemented a hospital-wide guideline focusing on reducing the use of high-risk antibiotics during the CDAD NAP1 epidemic in Quebec, Canada [16]. In this study, several interventions were implemented and evaluated in a time series analysis before, during, and after the peak epidemic period. After improved infection control procedures failed to have an impact on CDAD rates, local guidelines designed to reduce the use of high risk antibiotics and duration of therapy for pneumonia and intra-abdominal infections were developed by ID physicians and pharmacists and presented orally and via a pocket guide to all physicians and pharmacists. In the surveillance period after guideline implementation, targeted antibiotic use decreased 54% and CDAD rates declined by 60%. Although this study was conducted during an epidemic period when physicians were highly motivated to change, it suggests that nonrestrictive, educational interventions may be effective at reducing rates of CDAD under certain circumstances. 

It is important to note that the antibiotic restrictive and non-restrictive approaches are often not mutually exclusive and are implemented simultaneously in many cases. This comprehensive bundle approach is often more common in outbreak and epidemic settings. In a study by Muto and colleagues during an outbreak at the University of Pittsburg Medical Center, several interventions targeted at controlling the outbreak were implemented simultaneously. This CD bundle consisted of education, increased early CDAD case finding, improved infection control and development of a CD and antimicrobial management team which restricted high risk antimicrobial use. The overall impact of this CD bundle was a 71% decrease over the seven year period [17]. In assessing the long-term effects of ASP, Cook and Gooch evaluated several interventions including introduction of an ASP, implementation of electronic medical records, automated ASP alerts and restriction of ciprofloxacin and clindamycin over a 13-year period. The result of these interventions as a whole was a 42.6% reduction in hospital-acquired CD [18]. 

One unique strategy used in two separate studies by Delmee and colleagues and Johnson and colleagues was the treatment of asymptomatic carriers of CD [19,20]. Delmee *et al*. performed a quasi-experimental pre-post study in patients with leukemia in which patients with either symptomatic or asymptomatic CD were treated with oral vancomycin for seven days. Overall, the rate of positive CD toxin assays decreased from 9.9% to 1.2% in asymptomatic carriers. It is important to note that infection control efforts were occurring simultaneously and may have affected these results [20]. Johnson *et al.* treated 30 asymptomatic carriers of CD with vancomycin, metronidazole or placebo for 10 days and found that vancomycin initially cleared CD from 9/10 stools, performing better than both metronidazole (3/10) and placebo (2/10). However, the rate of recolonization approximately 20 days after treatment cessation was 89% [19]. Although asymptomatic carriers of CD have the potential to contribute to the horizontal spread of CD within an institution, antibiotic treatment of asymptomatic carriers is currently not supported by current guidelines. 

Both restrictive and nonrestrictive strategies to combat CDAD have been implemented by ASPs nationally and internationally and have had proven benefit at reducing the incidence of CDAD between 43%–71%. The use of ASPs with regards to CDAD must not be static but must be a dynamic process that is constantly be reassessed. As with any infectious disease managed by ASPs, the technologies and techniques utilized will need to continue to advance to stay ahead of the epidemic of CDAD and continue to improve patient outcomes. The following sections aim to highlight potential future directions for ASPs in the fight against CDAD that may be added to the tactics that have already documented success. 

## 2. Future Directions

Traditionally, ASPs have been primarily focused on individual antimicrobial agents and the overall optimization of antibiotic usage within the healthcare system. As the current focus of ASPs shifts towards total disease state management, a multifaceted approach that involves more than just the therapeutic pharmacologic agent is necessary. Antimicrobial stewardship must become a term that encompasses all interventions performed, established and executed by healthcare institutions with the goal of improving patient outcomes. As such, a global approach should be developed by ASPs that involves the prevention and treatment of disease acquisition, transmission and recurrence in all settings, both inpatient and outpatient. This method is especially crucial in combating CDAD given its predilection for person-to-person spread and the long term morbidity associated with multiple recurrences. The following sections explore future opportunities for ASPs to maximize their reach and breadth of effect against CD. 

### 2.1. Prescriber Restriction

As discussed, although antimicrobial restriction by ASPs has been the most successful intervention in reducing the rates of CDAD to date, it most often requires prescribers to call for approval of certain restricted antimicrobials prior to prescribing. This process places the burden of ensuring and “policing” the use of antimicrobials on the already overstretched ASP. Even with a labor-intensive restriction process in place, additional prospective audit and feedback is often required as prescribers may not be aware or may disregard the policy and they are usually permitted to order first doses of antibiotics during off hours. Although restricting the act of prescribing has been successful in many disease states including CD, restricting the prescribers themselves has yet to be explored. Several experts in the field of antimicrobial stewardship have proposed strategies that would restrict the ability to prescribe antibiotics to those prescribers who are uniquely qualified to understand both the individual patient risks and collateral damage associated with prescribing antimicrobials [21,22]. In a recent article, 72.6% of CD experts surveyed in Europe agreed that antibiotics should not be prescribed without the approval of a professional specifically trained in antimicrobial prescribing [23]. The framework for implementing these antimicrobial prescribing and stewardship competencies for prescribers has been laid out in a separate article [24]. This prescriber restriction program would be analogous to those already in place for prescribers of chemotherapy and would allow ASP programs to focus their efforts on novel interventions such as those discussed in the following sections of this manuscript. 

### 2.2. Rapid Diagnostics

The advancement in rapid diagnostic technologies has proven to be a powerful tool in the hands of ASPs. Molecular based technologies have been shown to have a dramatic impact on both patient outcomes and costs in important disease states such as *Staphylococcus aureus* and enterococcal bacteremia and *Acinetobacter baumannii* pneumonia [25]. To date, there are no published reports of combined rapid diagnostics and ASP interventions for the treatment or prevention of CDAD. This section reviews the currently available molecular technologies for identifying CD and discusses future opportunities for the use of these tests by ASPs. 

A recent expert review by Bassetti and colleagues and Collins and colleagues details the advances in our ability to identify and diagnose CD from stool samples [26,27]. As we have moved from CD toxin detection by enzyme immunoassay and glutamate dehydrogenase antigen detection to molecular biology studies, the sensitivity and specificity of these tests has increased dramatically. These molecular methods have also afforded the ability to perform both rRNA gene complex identification and ribotyping for infection control and diagnostic purposes. Certain machines in this category, like the Cepheid Xpert^®^ C. difficile/Epi machine have the ability to differentiate the NAP1 toxigenic strains from other strains of CD [28]. Although the association between the NAP1 strain and both severity of CD disease and treatment outcomes remains unclear [29], there are data to suggest that oral vancomycin may improve outcomes compared to fidaxomicin when treating patients with this strain [30,31]. This information could be used in real time by ASPs to designate treatment algorithms and allow for targeted, patient- and ribotype-specific interventions within one hour of sampling [32,33]. The benefit of rapid detection of CD via molecular methods also allows ASPs to recommend more rapid patient isolation and prevention of further patient-to-patient transmission of CD. In hospitals with high NAP1 prevalence detected via this machine, ASP practices with demonstrated effectiveness against this strain could be implemented, such as flouroquinolone restriction [34,35]. 

As more data becomes available on the impact of CD strain type on diseases severity and outcomes, routine strain typing may potentially become commonplace in the clinical arena. Several methods for typing currently exist including pulsed field gel electrophoresis, restriction endonuclease analysis and multilocus sequencing typing, although none of these methods are currently practical for routine use [27]. A new exciting technology introduced by Bomers and colleagues uses a portable mass spectrometry instrument called a field asymmetric ion mobility spectrometry (FAIMS) to quickly analyze the chemical composition of a stool sample and identify true CD with a sensitivity and specificity of 92.3% and 86%, respectively [36]. 

ASPs must recognize that the increased sensitivity of these molecular based tests will increase the detection rate of both true CD infection as well as colonization [37]. It is important to educate hospital administrators about these tests and the expected subsequent increase in the number of cases of CD, especially since hospital-acquired CD is now a publicly reported event in many states [38]. Finally, these tests can come with high costs, low clinical specificity and the inability to detect some variant strains of CD. Therefore, although promising these tests should be used judiciously until more data on patient outcomes and costs are reported by ASPs in order to justify the use of these instruments.

### 2.3. Treatment Paradigm

Recently the discussion regarding a shift away from metronidazole as a preferred treatment for CDAD has gained increased momentum [39,40,41]. This is due to the increased perturbation of the normal microbiome by metronidazole compared to oral vancomycin and fidaxomicin [42,43], clinical data that suggests inferiority when compared to oral vancomycin [44], and reports of treatment failures with metronidazole [45,46]. As oral vancomycin begins to replace metronidazole, fidaxomicin also continues to be recommended earlier and more often in treatment algorithms by experts in the field of CD, especially for recurrent CDAD [26,47]. Unfortunately, official guidelines from the Society of Healthcare Epidemiology of America (SHEA) and the Infectious Diseases Society of America (IDSA) lag behind the currently available data [48]. Despite being published in 2013, the American College of Gastroenterology Guidelines do not recommend fidaxomicin [49] while the 2014 European Society of Clinical Microbiology and Infectious Disease Guidelines include it as second line for non-severe disease [50]. 

In addition to initial FDA registration trials [30,31], fidaxomicin has proven to be useful in the real-world setting [51], as salvage therapy for patients with multiple recurrences [52], and in high-risk patient population including those with hematologic malignancies [53] and inflammatory bowel disease [54]. ASPs will need to consider the economic impact of increased utilization of fidaxomicin as its cost effectiveness in both initial treatment and recurrences compared to other treatment modalities has been disputed [55,56,57].

The clinical pipeline for both antibiotic and non-antibiotic therapies has reviewed in detail by Jarrad and colleagues [58]. Cadazolid (ACT179811; Actelion) is a novel protein synthesis inhibitor antimicrobial that possesses features of both the oxazolidinone and fluoroquinolone classes and has shown potent *in vitro* activity against CD [59]. Surotomycin (CB-183, 315; Merck) is an oral lipopeptide antibiotic structurally related to daptomycin shown to have potent efficacy in a hamster model of CDAD [60]. Regrettably, cadazolid and surotomycin are currently the only antibiotic therapies in phase III clinical trials for the treatment of CDAD (NCT01987895; NCT01598311 clinicaltrials.gov). SMT19969 (Summit Therapeutics), is a novel narrow-spectrum, non-absorbable antimicrobial shown to also have potent activity against CD with little activity against normal intestinal flora and is in currently in phase II clinical trials (NCT02092935) [61]. 

Fecal bacteriotherapy continues to gain increased attention as a reliable treatment option for CDAD, especially in patients with multiple disease recurrences. Originally administered as fecal microbiota transplant from donors via nasogastric tube, enema, or colonoscopy, success rates as high as 92% were reported. Despite this reported success, the invasiveness and need for an individual donor per patient precludes its routine use in the inpatient setting and for patients who are critically ill with CDAD [62]. A recently introduced alternative using frozen fecal suspensions from pooled donors administered via nasogastric tube or by capsule has shown similar success rates and greater feasibility [63,64]. These original homogenized fecal suspension techniques have since been refined to examine the efficacy of only certain bacterial strains on treating and preventing CDAD. These include treatment with nontoxigenic strains of CD designed to outcompete toxigenic strains and prevent colonization, species other than *C. difficile* including *C. scindens*, and probiotics such as *Lactobacillus acidophilus and casei*, several of which are currently in phase II clinical trials (NCT01259726 and NCT01925417). An excellent review on the role of bacteriotherapy in the treatment of a host of gastrointestinal conditions, including CDAD, has been recently published and also discusses positive results for the probiotics *Saccharmyces boulardii and L. rhamnosus* GG [65] along with a meta-analysis and systematic review in this journal [66].

Preventative treatments for CDAD are also being increasingly explored. An anti-CD vaccine (PF-06425090) being developed by Sanofi Pasteur has shown promise and is currently in phase III trials (NCT01887912). Additionally, secondary prophylaxis with oral vancomycin for patients with a history of CDAD who are at high risk of recurrence has demonstrated a 36% reduction in the rate of CDAD acquisition [67].

ASPs will need to continually monitor the ever-changing treatment landscape for CD and evaluate individual prevention and treatment strategies according to the needs of their institutions. The more pervasive use of fidaxomicin and advent of advanced recombinant fecal microbiota therapies will certainly put financial pressure on ASPs, and cost justification of these strategies in light of their improved clinical outcomes will be required. ASPs must work closely with hospital administration in order to ensure the ASP is positioned to demonstrate value and recognize that part of the mission of both the ASP and the C-suite is to reduce the global cost of care [68]. The understanding that the additional drug costs incurred through the use of a more expensive therapeutic regimen that has demonstrated improved patient outcomes is ultimately a cost effective proposition for both groups is paramount. 

### 2.4. Infection Control

Environmental contamination rates of CD have been reported as high as 58% [69] and previous room occupants’ CD status is a significant risk for development of CDAD (hazard ratio 2.35, *p =* 0.01) [70]. Preventing the horizontal transmission of CD represents a major challenge for healthcare institutions primarily due to the ability of CD to survive in its vegetative state as a spore and resist typical hospital cleaning methods, including alcohol based hand sanitizers [71,72]. These spores can persist on surfaces even after terminal room cleaning and retain the ability to germinate and cause CDAD [70]. While hand hygiene remains the most tried and true measure to prevent healthcare-associated infections, more reliable, technologically advanced strategies are needed to combat CDAD [73]. 

A host of successful infection control strategies have been recommended and are currently utilized to reduce the spread of CD within the healthcare institution. These strategies include CD case surveillance, barrier precautions, patient isolation and cohorting, use of disposable medical devices and environmental disinfection. All of these methods have shown a significant reduction in the rates of CD transmission, with the exception of patient isolation and cohorting, and are highlighted in the review by Hsu and colleagues [74]. Despite these strategies, the rates of healthcare-associated infections due to CD continue to increase [75,76]. In response to this challenge, several new technologies have emerged to add to the armamentarium of infection controls tools.

The most recent advances in combating the transmission of CD within the healthcare system have come in the form of automated room and surface disinfecting technologies. The first of these technologies is portable pulsed xenon ultraviolet (UV) light robots, which use high-intensity broad-spectrum UV irradiation in the 200–320 nm range to break the molecular bonds in DNA of CD, thereby destroying the organism and its spores [77,78]. Compared to traditional UV radiation devices that use low pressure mercury gas bulbs, the pulsed xenon UV produces a broader spectrum of radiation emitted in high intensity pulses. This machine has been shown to produce a 53% reduction in CD infections even after terminal room cleaning is complete and is approximately 22% faster than manual cleaning. In a retrospective study, pulsed xenon UV disinfection after terminal room cleaning decreased multi-drug resistant organism and CD rates by 20% over a 22 month period [79]. This technology has also been shown to reduce recovery of CD spores on glass surfaces and frequently touched surfaces [80]. 

Another advancement in CDAD prevention is the fully automated advanced ultrasonic machine called the HJ-30i from Altapure [81]. The HJ-30i is a remotely operated machine which aerosolizes a dense cloud of sub-micron particles of a 0.88% hydrogen peroxide, 0.18% peroxyacetic aced and 98.6% purified water mixture. This cloud is capable of three dimensional high-level disinfection of large spaces producing a 6-log kill in less than 10 minutes. It has been shown to produce a 90% kill of *Geobacillus stearothermophilus*, a surrogate for CD, in under three minutes. From start to finish, it can help turnover a hospital room in as little as one hour. After use of the HJ-30i for 23 min in one hospital room, none of the inoculated surfaces showed any detectable growth of any of the five pathogens tested, including CD [82].

As opposed to the previous systems, the Health Risk Management System (HRMS) from Arcalux^®^ and American Green Technology^®^ is not a standalone portable robotic system [83]. The HRMS is a ceiling-mounted air disinfection system that doubles as a lighting fixture clinically proven to eliminate 99.7% of pathogens causing hospital-acquired infections. This system works unobtrusively and is designed as an air purification system that uses differential pressure to isolate airborne contaminants in the UV radiation chamber, eliminating 97% of pathogens in the first pass. The continuously operating HRMS cleans the air up to four times per hour and has a combined single pass removal and killing rate of 100% for CD spores, even at an inoculum up to 800 times the normal hospital level. The zonal protection factor (*i.e.*, the percentage of a given area able to be sterilized) for 100 square foot room was 91% [84].

ASPs are in a prime position to be both a promoter of and mediator not only for new drug therapies but emerging technologies that can be implemented to improve patient outcomes. ASPs often have an established relationship with infection control practitioners and can play a valuable role in the implementation and cost justification for new infection control measures, as CD incidence data is likely already being collected. Along with rapid diagnostic techniques, ASPs should play a significant part in lobbying for technologies that can improve their efficiency by reducing disease incidence and help them focus their time and effort into patient-level interventions. Despite the heterogeneity between outcome markers and the current lack of real-world clinical use, these advanced infection control mechanisms will continue to supplement and potentially replace manual environmental cleaning.

### 2.5. Public Awareness

Knowledge amongst the general public is crucial to combating difficult to treat and rapidly spreading infectious disease pathogens, especially those that cause a tremendous amount of morbidity such as CD. The ultimate goal of any ASP should be to make everyone a better steward; this includes patients, their families, and the general public. Organizations such as the C Diff Foundation (cdifffoundation.org) strive to support public health education and advocate for CD infection prevention, treatments, and environmental safety around the world. The C Diff Foundation is a nonprofit organization founded in 2012 whose volunteers host a 24 h hotline to support providers and patients in managing the difficulties of CD infection in 20 countries around the world. Their mission is to bring positive results in the reduction in the number of newly diagnosed cases of CD through collaborative efforts from healthcare providers, corporate organizations, government agencies, employers and communities. ASPs should work to align themselves with organizations like the C Diff Foundation in order to ignite change and spread the reach of their efforts outside the healthcare organization. A concerted effort should be made by ASPs to inform the general public of the risk factors, signs and symptoms, contagiousness and availability of support and advocacy groups for CDAD. 

### 2.6. Outpatient ASP

The majority of prescriptions for antimicrobials are written for patients in the outpatient setting where overuse and misuse are common. Cases of CDAD have been documented in outpatient healthcare facilities including hemodialysis centers [85] and in patients on outpatient parenteral antimicrobial therapy [86]. A recent review evaluating the effect of outpatient ASPs demonstrated that many of the same interventions discussed in this review, including prescriber feedback/communication and diagnostic testing, are effective in reducing antimicrobial overuse and improving prescribing in the non-acute care setting [87]. Although patient level outcomes were rarely reported in this review, the majority of antimicrobials examined were prescribed for respiratory tract and skin and soft tissue infections, two areas where high-risk CDAD antibiotics are often utilized. ASPs must begin to expand their services within the healthcare system in order to reach patients outside the acute care setting in order to combat non-nosocomially acquired CDAD. A recently developed predictive score can help ASPs identify high risk outpatients and implement targeted interventions [88]. The need for outpatient ASP interventions targeted at CDAD also encompasses infection control as surveillance estimates that include only inpatient CDAD cases can miss >80% of the total number of cases [89]. Furthermore, patients diagnosed with CDAD as an inpatient who have follow up visits in the clinic afterwards have been shown to contaminate the outpatient environment with CD spores [90]. Finally, a focus on transitions of care is also vitally important as previous stay in a healthcare setting has been shown to be an independent predictor of identifying CDAD in the outpatient setting [91]. 

## 3. Discussion

The role of antibiotic stewardship in improving patient care and health outcomes has been well documented [92,93], including the impact of ASPs on CDAD. An algorithm for the prevention of nosocomially-acquired CDAD has recently been proposed by a panel of CD experts, recommending ASP to minimize host susceptibility to CDAD as in integral part of a multidimensional effort to combat this pathogen [94]. Stewardship approaches that restrict antimicrobials, especially those that have been associated with a high risk of CDAD, have proven to be the most successful at reducing CD incidence. Nonrestrictive policies, such as prospective audit and feedback and implementation of new hospital procedures or guidelines, have also demonstrated success, albeit often when combined with restrictive strategies. 

CDAD is a multifactorial problem which requires a multifaceted global solution, including optimizing antimicrobial use, improved diagnostics for identification of CD, development of new antibiotic and non-antibiotic treatment regimens, optimized infection control measures and novel educational activities for healthcare professionals and the public. To date, ASPs have proven to be the most useful tool in the fight against CDAD, but more can be done. A unique niche has been created to either encompass or play a major role in each one of the suggested future intervention areas, and these opportunities should be exploited. Antimicrobial stewardship is a broad sweeping term used for all activities performed, developed and implemented by healthcare institutions with the aim of decreasing inappropriate antibiotic use, preventing the spread of bacterial resistance, and improving patient outcomes. This includes the prevention and treatment of hospital-associated infections such as CD. 

## 4. Conclusions

As CDAD continues to be a significant cause of morbidity and mortality in patients and remain a dangerous public health threat, ASPs will need to develop new tactics to improve patient outcomes. We have outlined several potential avenues which should be explored by ASPs including prescriber restrictions, emerging rapid diagnostics, innovative treatment strategies, advanced infection control technologies and promoting public awareness. Along with continuing to implement and improve on existing interventions, several exciting strategies are on the horizon for the prevention and treatment of CDAD. 
